# Supplementary material for: An empirical Bayes approach to stochastic blockmodels and graphons: shrinkage estimation and model selection
Source: PeerJ Comput Sci. 2022 Jul 6;8:e1006. doi: 10.7717/peerj-cs.1006 (PMC9299287; doi:10.7717/peerj-cs.1006)
Supplement: Supplemental Information 1 [file peerj-cs-08-1006-s001.pdf]

SUPPLEMENTARY MATERIAL

VARIATIONAL BAYES EM

As an extension to the work in Daudin et al. [2008], Latouche et al. [2012] proposed a variational Bayes approximation to provide a closed form approximate posterior distribution of the parameters (π, Θ) and of the latent variables $\mathbf{Z}$, where the observed-data log-likelihood can be decomposed into two terms,

$$\ln p(\mathbf{X}) = \mathscr{L}(q(\cdot)) + \mathrm{KL}(q(\cdot)\|p(\cdot|\mathbf{X})), \tag{S1}$$

where

$$\mathscr{L}(q(\cdot)) = \sum_{\mathbf{Z}} \int\int q(\mathbf{Z}, \pi, \Theta) \ln\left\{\frac{p(\mathbf{X}, \mathbf{Z}, \pi, \Theta)}{q(\mathbf{Z}, \pi, \Theta)}\right\} d\pi d\Theta, \tag{S2}$$

and

$$\mathrm{KL}(q(\cdot)\|p(\cdot|\mathbf{X})) = -\sum_{\mathbf{Z}} \int\int q(\mathbf{Z}, \pi, \Theta) \ln\left\{\frac{p(\mathbf{Z}, \pi, \Theta|\mathbf{X})}{q(\mathbf{Z}, \pi, \Theta)}\right\} d\pi d\Theta. \tag{S3}$$

Minimizing (S3) with respect to $q(\mathbf{Z}, \pi, \Theta)$ is equivalent to maximizing the lower bound (S2) with respect to $q(\mathbf{Z}, \pi, \Theta)$. However, when considering SBM, $q(\mathbf{Z}, \pi, \Theta)$ is intractable, thus we can assume that it can be factorized as

$$q(\mathbf{Z}, \pi, \Theta) = q(\mathbf{Z})q(\pi)q(\Theta) = q(\pi)q(\Theta)\prod_{i=1}^{N} q(z_i), \tag{S4}$$

where the optimal approximation $q(z_i)$ at vertex i follows a multinomial distribution. Latouche et al. [2012] used a variational Bayes EM (VBEM) algorithm described in Beal and Ghahramani [2003] to optimize over $q(z_i)$ and $q(\pi)$, $q(\Theta)$ iteratively.

SUPPLEMENTARY FIGURES AND TABLES

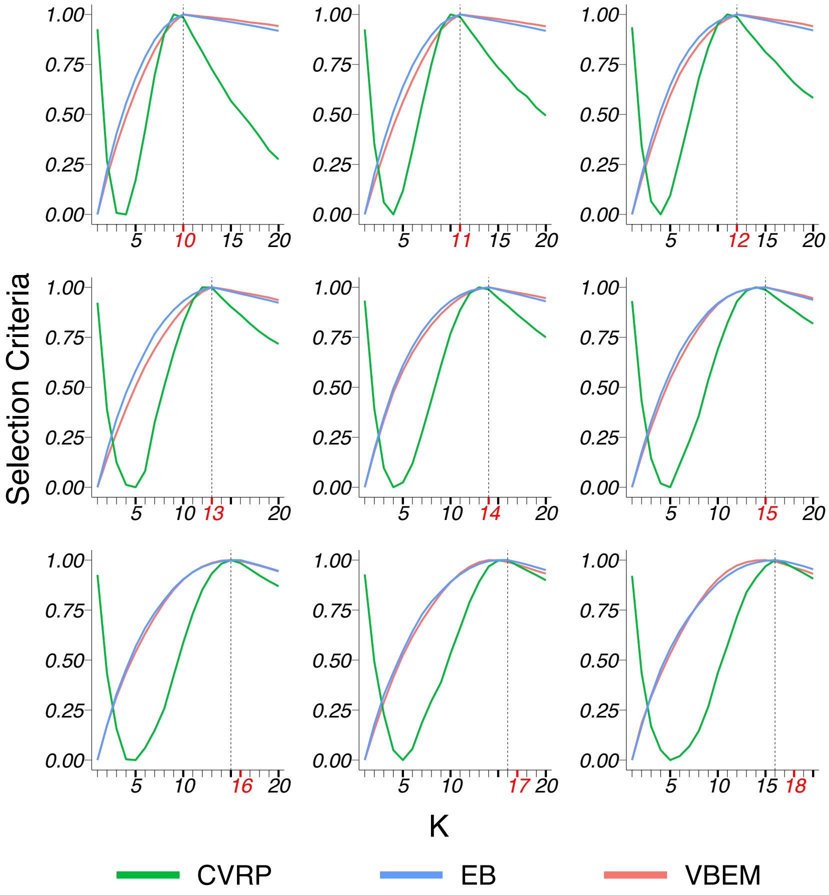

Figure S1 – Values of model selection criteria in model 1 simulation. The true number of blocks K^* (marked as red) ranges from 10 to 18 and the results for graphs with each K^* are shown in a panel. $\mathscr{I}_{\mathrm{CVRP}}$, $\mathscr{I}_{\mathrm{VBEM}}$, $\mathscr{I}_{\mathrm{EB}}$ are all standardized to $[0,1]$, and $\mathscr{I}_{\mathrm{CVRP}}$ is taken negative, thus the model is selected by the maximizer of each criterion. For the 100 graphs generated under each set of parameters, the values of three criteria are plotted against the input number of blocks $K = 1,\ldots,20$ used in clustering. The number of clusters selected by EB is highlighted by the dashed lines.

Table S1 – MSE values for model 1. The average MSE of the estimates $\widehat{\Theta}$ to Θ by the three methods of the 100 graphs generated under different K^* are shown in each row. The input number of clusters K ranges from 10 to 18, for each K^* and K the minimal MSE among three methods is boldfaced.

$K^* \setminus K$		9	10	11	12	13	14	15	16	17	18	19	20	
	MLE	304	25	35	46	56	67	77	93	109	120	131	148	$\times 10^{-5}$
10	VBEM	304	25	36	46	55	66	76	90	106	116	127	143	$\times 10^{-5}$
	EB	**289**	**2**	**32**	**42**	**50**	**59**	**65**	**75**	**89**	**94**	**105**	**117**	$\times 10^{-5}$
	MLE	538	249	30	41	**52**	60	72	85	96	112	123	139	$\times 10^{-5}$
11	VBEM	538	249	31	41	**52**	60	72	85	95	111	120	136	$\times 10^{-5}$
	EB	**522**	**229**	**3**	**34**	54	**56**	**66**	**76**	**83**	**94**	**100**	**111**	$\times 10^{-5}$
	MLE	683	417	215	35	44	55	**67**	79	89	99	112	130	$\times 10^{-5}$
12	VBEM	683	417	216	36	45	56	**67**	79	89	100	112	129	$\times 10^{-5}$
	EB	**668**	**398**	**192**	**3**	**31**	**54**	70	**76**	**81**	**86**	**97**	**107**	$\times 10^{-5}$
	MLE	1012	708	434	212	41	51	**62**	**75**	**85**	**98**	111	128	$\times 10^{-5}$
13	VBEM	1012	708	434	212	42	52	63	76	86	99	112	128	$\times 10^{-5}$
	EB	**996**	**689**	**410**	**183**	**3**	**36**	65	85	91	100	**103**	**111**	$\times 10^{-5}$
	MLE	921	692	487	305	167	48	60	71	81	**91**	**102**	**114**	$\times 10^{-5}$
14	VBEM	921	692	488	305	168	49	61	73	82	93	104	116	$\times 10^{-5}$
	EB	**906**	**673**	**464**	**276**	**133**	**3**	**43**	**65**	**79**	94	**102**	**114**	$\times 10^{-5}$
	MLE	969	733	543	389	262	149	57	68	78	**88**	**99**	**114**	$\times 10^{-5}$
15	VBEM	969	733	543	390	263	150	58	70	81	91	102	116	$\times 10^{-5}$
	EB	**953**	**712**	**518**	**359**	**227**	**108**	**5**	**43**	**69**	89	104	120	$\times 10^{-5}$
	MLE	1044	842	653	495	361	237	137	70	77	89	101	**114**	$\times 10^{-5}$
16	VBEM	1044	842	653	495	362	238	138	72	80	92	104	117	$\times 10^{-5}$
	EB	**1028**	**822**	**629**	**466**	**326**	**197**	**91**	**16**	**43**	**74**	**97**	115	$\times 10^{-5}$
	MLE	1132	907	705	541	388	264	190	124	124	125	137	146	$\times 10^{-5}$
17	VBEM	1132	907	705	541	389	265	191	125	126	128	140	149	$\times 10^{-5}$
	EB	**1116**	**887**	**681**	**512**	**354**	**224**	**143**	**70**	**82**	**102**	**129**	**144**	$\times 10^{-5}$
	MLE	1097	905	733	583	458	348	247	161	137	142	141	164	$\times 10^{-5}$
18	VBEM	1097	905	733	583	458	348	248	162	139	144	144	167	$\times 10^{-5}$
	EB	**1082**	**886**	**709**	**553**	**423**	**307**	**199**	**107**	**81**	**96**	**104**	**143**	$\times 10^{-5}$

Table S2 – MSE values for model 2. The average MSE of the estimates $\widehat{\Theta}$ to Θ by the three methods of the 100 graphs generated under different n are shown in each row. The input number of clusters K ranges from 1 to 12, for each n and K the minimal MSE among three methods is boldfaced.

$n \setminus K$		1	2	3	4	5	6	7	8	9	10	11	12	
	MLE	**229**	225	225	229	236	238	247	256	266	269	273	283	$\times 10^{-5}$
200	VBEM	**229**	**221**	**219**	**222**	**225**	**230**	**236**	**240**	251	255	260	269	$\times 10^{-5}$
	EB	**229**	224	224	227	233	233	239	243	**250**	**250**	**251**	**258**	$\times 10^{-5}$
	MLE	**230**	217	204	192	186	182	184	187	192	193	202	206	$\times 10^{-5}$
250	VBEM	**230**	**215**	**201**	**188**	**181**	**176**	**177**	**181**	184	186	194	198	$\times 10^{-5}$
	EB	**230**	217	203	192	185	180	180	**181**	**182**	**182**	**187**	**190**	$\times 10^{-5}$
	MLE	**231**	**208**	186	165	147	130	121	120	118	124	128	133	$\times 10^{-5}$
300	VBEM	**231**	**208**	**185**	**164**	**145**	**128**	**119**	**117**	114	120	123	128	$\times 10^{-5}$
	EB	**231**	**208**	186	165	147	130	120	**117**	**113**	**117**	**119**	**123**	$\times 10^{-5}$
	MLE	**231**	**202**	175	151	129	110	94	81	74	75	77	77	$\times 10^{-5}$
350	VBEM	**231**	**202**	**174**	**150**	**128**	**109**	**93**	**80**	**73**	73	76	76	$\times 10^{-5}$
	EB	**231**	**202**	175	**150**	**128**	110	**93**	**80**	**73**	**72**	**73**	**72**	$\times 10^{-5}$
	MLE	**232**	**201**	171	**141**	**117**	95	**77**	61	49	44	43	45	$\times 10^{-5}$
400	VBEM	**232**	**201**	**170**	**141**	**117**	95	**77**	**60**	48	44	42	44	$\times 10^{-5}$
	EB	**232**	**201**	**170**	**141**	**117**	**94**	**77**	**60**	**47**	**43**	**41**	**42**	$\times 10^{-5}$
	MLE	**232**	**199**	**167**	**139**	**114**	**91**	**70**	52	36	25	26	28	$\times 10^{-5}$
450	VBEM	**232**	**199**	**167**	**139**	**114**	**91**	**70**	52	36	25	26	27	$\times 10^{-5}$
	EB	**232**	**199**	**167**	**139**	**114**	**91**	**70**	**51**	**35**	**24**	**24**	**26**	$\times 10^{-5}$

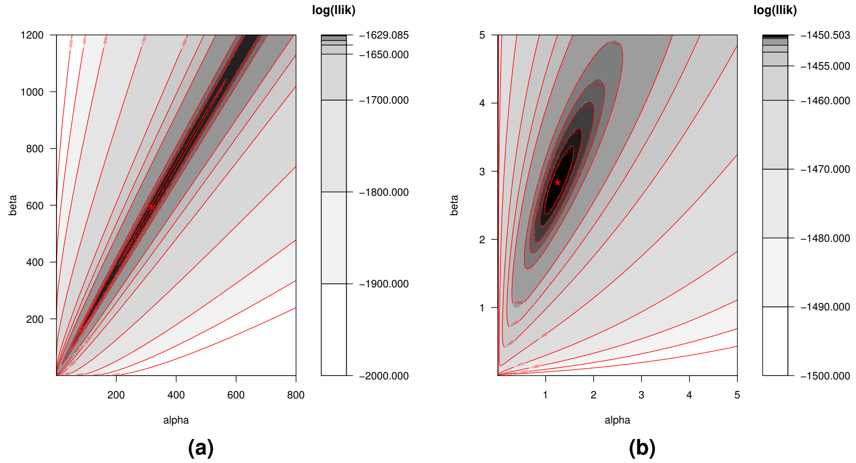

Figure S2 – A typical contour plot of (a) $\mathscr{L}(\alpha_0, \beta_0)$ and (b) $\mathscr{L}(\alpha_1, \beta_1)$ from a graph generated by a SBM with $n = 200$, $K = 5$, $\theta_{ab} = 0.7$ for $a = b$ and $\theta_{ab} = 0.3$ for $a \neq b$. The maximizers are marked as stars in the plots.

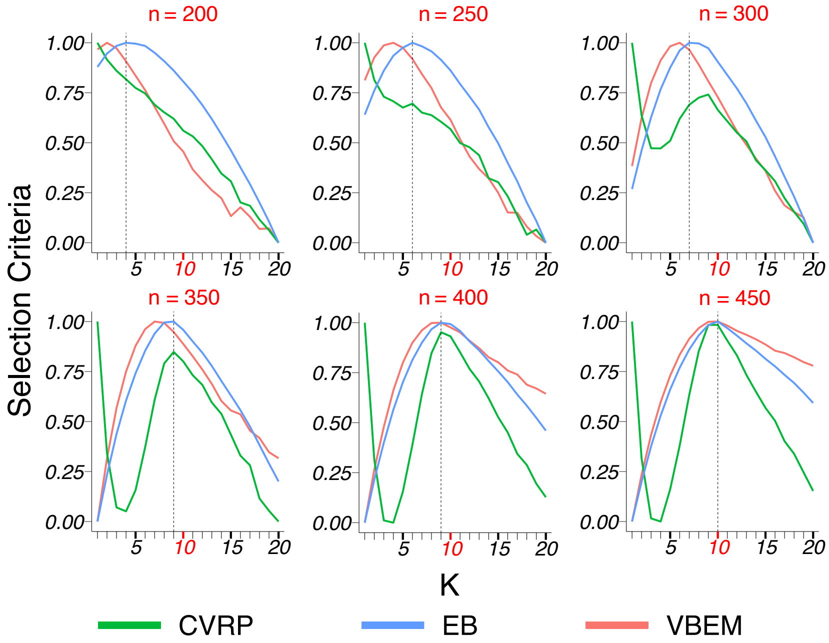

Figure S3 – Values of model selection criteria in model 2 simulation. With the true number of blocks $K^* = 10$ (marked as red), the number of network sizes n ranges from 200 to 450 and the results for graphs with each n are shown in a panel. $\mathscr{J}_{\text{CVRP}}$, $\mathscr{J}_{\text{VBEM}}$, $\mathscr{J}_{\text{EB}}$ are all standardized to $[0,1]$, and $\mathscr{J}_{\text{CVRP}}$ is taken negative, thus the model is selected by the maximizer of each criterion. For the 100 graphs generated under each set of parameters, the values of three criteria are plotted against the input number of blocks $K = 1, \ldots, 20$ used in clustering. The number of clusters selected by EB is highlighted by the dashed lines.

Table S3 – MSE values for model 3. The average MSE of the estimates $\widehat{\Theta}$ to Θ by the three methods of the 100 graphs generated under different set of parameters ρ and λ are shown in each row. The input number of clusters K ranges from 1 to 10, for each ρ, λ and K the minimal MSE among three methods is boldfaced.

ρ	λ		1	2	3	4	5	6	7	8	9	10	
10^{-1}	2	MLE	**80**	**26**	28	34	43	47	57	70	80	91	$\times 10^{-4}$
		VBEM	81	28	31	46	65	78	92	103	114	120	$\times 10^{-4}$
		EB	**80**	**26**	**26**	**31**	**38**	**40**	**46**	**54**	**58**	**62**	$\times 10^{-4}$
	3	MLE	**229**	78	**47**	50	57	62	73	83	94	104	$\times 10^{-4}$
		VBEM	248	109	75	90	110	130	147	163	177	191	$\times 10^{-4}$
		EB	**229**	**77**	**47**	**48**	**53**	**58**	**66**	**73**	**79**	**84**	$\times 10^{-4}$
	5	MLE	**680**	**247**	**161**	**144**	**144**	148	153	164	171	184	$\times 10^{-4}$
		VBEM	806	529	451	452	483	517	555	581	612	638	$\times 10^{-4}$
		EB	**680**	248	**161**	145	**144**	**145**	**150**	**158**	**163**	**171**	$\times 10^{-4}$
$10^{-1.5}$	2	MLE	**8**	**11**	**13**	14	17	18	21	22	24	25	$\times 10^{-4}$
		VBEM	10	20	59	98	133	169	204	234	269	305	$\times 10^{-4}$
		EB	**8**	**11**	**13**	**13**	**15**	**14**	**14**	**15**	**16**	**16**	$\times 10^{-4}$
	3	MLE	**23**	**13**	16	19	24	28	32	34	40	42	$\times 10^{-4}$
		VBEM	32	25	63	102	132	166	202	236	265	304	$\times 10^{-4}$
		EB	**23**	**13**	**15**	**15**	**18**	**19**	**21**	**21**	**23**	**23**	$\times 10^{-4}$
	5	MLE	**68**	**30**	32	35	40	45	54	64	70	88	$\times 10^{-4}$
		VBEM	127	132	166	213	258	301	339	369	415	452	$\times 10^{-4}$
		EB	**68**	**30**	**30**	**31**	**33**	**36**	**39**	**42**	**45**	**49**	$\times 10^{-4}$
10^{-2}	2	MLE	**82**	190	239	336	346	429	425	442	498	490	$\times 10^{-6}$
		VBEM	58	795	1525	2183	2821	3461	4098	4705	5248	5799	$\times 10^{-5}$
		EB	**82**	**187**	**185**	**232**	**273**	**224**	**219**	**192**	**202**	**192**	$\times 10^{-6}$
	3	MLE	**23**	44	53	58	68	71	68	73	74	67	$\times 10^{-5}$
		VBEM	127	889	1644	2397	3103	3814	4509	5164	5774	6413	$\times 10^{-5}$
		EB	**23**	**43**	**47**	**48**	**53**	**52**	**52**	**53**	**54**	**43**	$\times 10^{-5}$
	5	MLE	**68**	**136**	153	160	173	165	174	171	160	166	$\times 10^{-5}$
		VBEM	413	1129	1998	2941	3804	4667	5413	6215	6943	7586	$\times 10^{-5}$
		EB	**68**	142	**136**	**143**	**137**	**124**	**132**	**123**	**123**	**118**	$\times 10^{-5}$

Table S4 – MSE values for model 4. The average MSE of the estimates $\widehat{\Theta}$ to Θ by the three methods of the 100 graphs generated under different set of parameters ρ and λ are shown in each row. The input number of clusters K ranges from 1 to 10, for each ρ, λ and K the minimal MSE among three methods is boldfaced.

ρ	λ		1	2	3	4	5	6	7	8	9	10	
10^{-1}	2	MLE	**782**	**240**	**120**	77	82	84	87	114	112	121	$\times 10^{-5}$
		VBEM	783	244	122	78	121	177	228	270	327	374	$\times 10^{-5}$
		EB	**782**	**240**	**120**	**76**	**77**	**78**	**81**	**99**	**98**	**103**	$\times 10^{-5}$
	3	MLE	**225**	**69**	**33**	**20**	**15**	**14**	15	15	18	18	$\times 10^{-4}$
		VBEM	230	82	44	29	23	26	31	36	42	48	$\times 10^{-4}$
		EB	**225**	**69**	**33**	**20**	**15**	**14**	**14**	**14**	**16**	**16**	$\times 10^{-4}$
	5	MLE	**674**	**230**	**131**	**103**	**91**	**85**	**83**	**83**	84	85	$\times 10^{-4}$
		VBEM	719	379	268	242	234	232	234	241	248	257	$\times 10^{-4}$
		EB	**674**	**230**	**131**	**103**	**91**	**85**	**83**	**83**	**83**	**84**	$\times 10^{-4}$
$10^{-1.5}$	2	MLE	**78**	**24**	21	22	28	32	39	45	52	78	$\times 10^{-5}$
		VBEM	80	26	32	138	242	342	450	512	623	686	$\times 10^{-5}$
		EB	**78**	**24**	**20**	**21**	**23**	**25**	**27**	**32**	**31**	**37**	$\times 10^{-5}$
	3	MLE	**225**	**68**	**33**	37	43	52	65	84	96	125	$\times 10^{-5}$
		VBEM	244	102	62	151	248	323	416	455	517	559	$\times 10^{-5}$
		EB	**225**	**68**	**33**	**35**	**38**	**44**	**48**	**56**	**60**	**67**	$\times 10^{-5}$
	5	MLE	**674**	**213**	**102**	73	79	93	106	119	151	180	$\times 10^{-5}$
		VBEM	853	641	506	472	577	680	785	854	903	947	$\times 10^{-5}$
		EB	**674**	**213**	**102**	**72**	**73**	**79**	**86**	**96**	**112**	**125**	$\times 10^{-5}$
10^{-2}	2	MLE	**8**	8	15	15	17	18	20	22	20	18	$\times 10^{-5}$
		VBEM	10	20	176	305	476	626	778	915	1065	1250	$\times 10^{-5}$
		EB	**8**	**8**	**12**	**11**	**13**	**12**	**13**	**12**	**14**	**12**	$\times 10^{-5}$
	3	MLE	**23**	**9**	16	20	21	25	33	29	33	31	$\times 10^{-5}$
		VBEM	33	19	168	325	471	656	792	994	1121	1301	$\times 10^{-5}$
		EB	**23**	**9**	**11**	**11**	**14**	**14**	**15**	**15**	**18**	**17**	$\times 10^{-5}$
	5	MLE	**67**	**23**	35	39	44	60	70	88	105	130	$\times 10^{-5}$
		VBEM	124	120	196	358	523	655	829	984	1084	1277	$\times 10^{-5}$
		EB	**67**	**23**	**23**	**26**	**29**	**31**	**33**	**35**	**40**	**42**	$\times 10^{-5}$

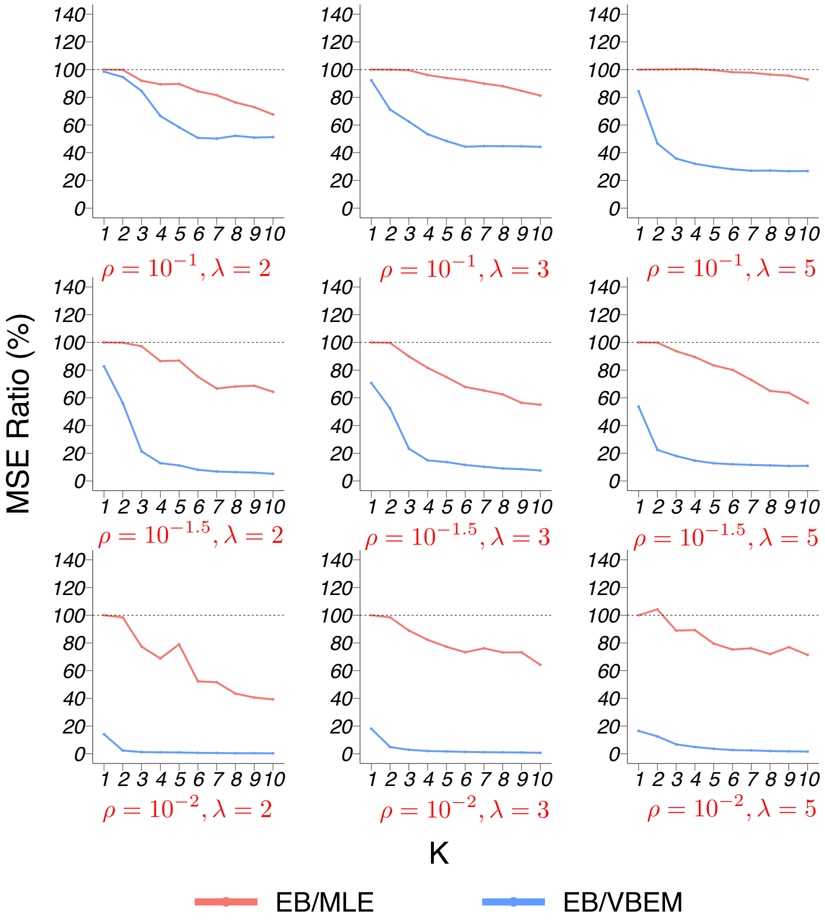

Figure S4 – MSE ratios in model 3 simulation with graph size $n = 100$. The results for graphs with each combination of ρ and λ are shown in a panel.

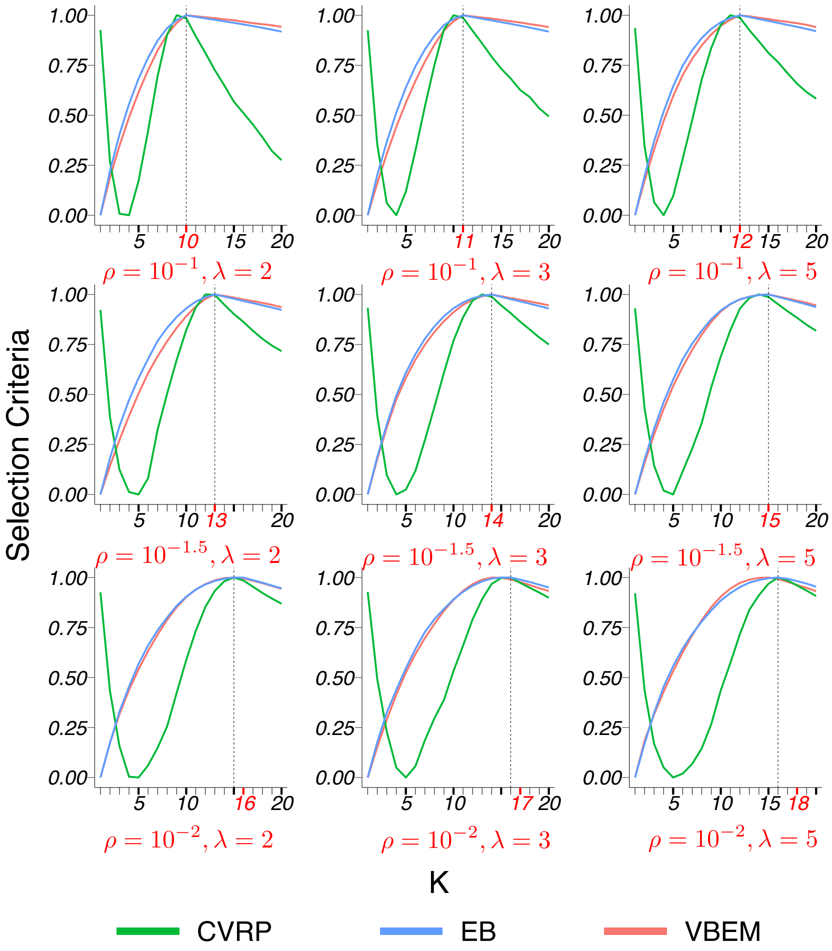

Figure S5 – Values of model selection criteria in model 3 simulation. Graph size (number of nodes) $n = 100$. With the graphon $W(x,y) = \rho\lambda^2(xy)^{\lambda-1}$, $\rho \in \{10^{-1}, 10^{-1.5}, 10^{-2}\}$ and $\lambda \in \{2,3,5\}$, the results for graphs with each set of parameters ρ and λ are shown in a panel. $\mathscr{J}_{\text{CVRP}}$, $\mathscr{J}_{\text{VBEM}}$, $\mathscr{J}_{\text{EB}}$ are all standardized to $[0,1]$, and $\mathscr{J}_{\text{CVRP}}$ is taken negative, thus the model is selected by the maximizer of each criterion. For the 100 graphs generated under each set of parameters, the values of three criteria are plotted against the input number of blocks $K = 1,\ldots,10$ used in clustering. The number of clusters selected by EB is highlighted by the dashed lines.

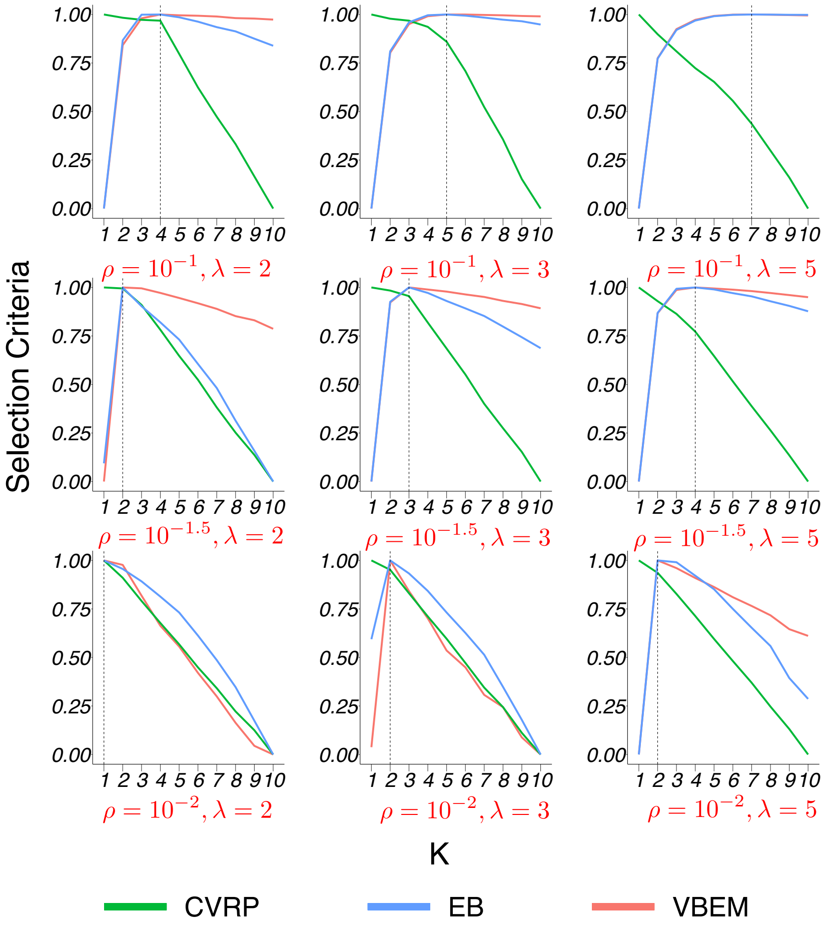

Figure S6 – Values of model selection criteria in model 4 simulation. Graph size (number of nodes) $n = 316$. With the graphon $W(x,y) = \rho\lambda^2(xy)^{\lambda-1}$, $\rho \in \{10^{-1}, 10^{-1.5}, 10^{-2}\}$ and $\lambda \in \{2,3,5\}$, the results for graphs with each set of parameters ρ and λ are shown in a panel. $\mathscr{J}_{\text{CVRP}}$, $\mathscr{J}_{\text{VBEM}}$, $\mathscr{J}_{\text{EB}}$ are all standardized to $[0,1]$, and $\mathscr{J}_{\text{CVRP}}$ is taken negative, thus the model is selected by the maximizer of each criterion. For the 100 graphs generated under each set of parameters, the values of three criteria are plotted against the input number of blocks $K = 1,\ldots,10$ used in clustering. The number of clusters selected by EB is highlighted by the dashed lines.

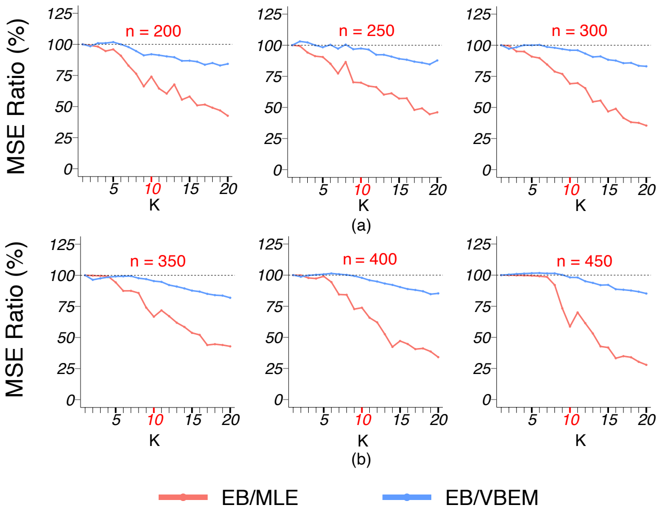

Figure S7 – MSE ratios in model 2s simulation. The true number of blocks K^* (marked in red) ranges from 10 to 18 and the results for graphs with each K^* are shown in a panel. For the 100 graphs generated under each K^*, the MSE ratios of the estimates $\widehat{\Theta}_{\text{MLE}}$ and $\widehat{\Theta}_{\text{VBEM}}$ over $\widehat{\Theta}_{\text{EB}}$ are plotted against the input number of blocks K chosen in the clustering step.

Table S5 – Model selection comparison for model 2s among the $\hat{K}$ chosen by (a) CVRP, (b) VEBM, and (c) EB. (Each row in a table reports the frequency of $\hat{K}$ across 100 graphs. The last two columns report two mean absolute deviations, the minimum of which among the three methods is in red for each K^*.

(a) CVRP

$n\backslash\hat{K}$	1	2	3	4	5	6	7	8	9	10	11	12	13	14	15	E_{K^*}	$E_{\hat{K}}$
200	100															9.00	0.20
250	100								5	20	27	10	3		1	9.00	1.11
300	99	1														8.99	3.62
350	99	1														8.99	7.14
400	100															9.00	9.25
450	89	1							2	8						8.11	10.44

(b) VBEM

$n\backslash\hat{K}$	1	2	3	4	5	6	7	8	9	10	11	12	13	14	15	E_{K^*}	$E_{\hat{K}}$
200	87	13														8.87	0.25
250	61	35	3	1					5	20	27	10	3		1	8.56	1.03
300	28	33	30	6	3											7.77	2.62
350	3	10	26	33	16	7	3	2								6.08	4.25
400	1	1	7	17	30	21	11	6	4	2						4.51	4.80
450					4	16	27	28	15	7	2		1			2.41	4.68

(c) EB

$n\backslash\hat{K}$	1	2	3	4	5	6	7	8	9	10	11	12	13	14	15	E_{K^*}	$E_{\hat{K}}$
200	96	4														8.96	0.24
250	91	7	2													8.89	1.04
300	17	78	3	1	1											7.69	2.42
350		4	15	23	37	8	5	3	3	2						5.16	4.09
400		1	2	2	10	17	18	8	8	9	5	12	11			2.92	4.67
450							2	3	11	13	17	11	14	16	13	2.33	4.06

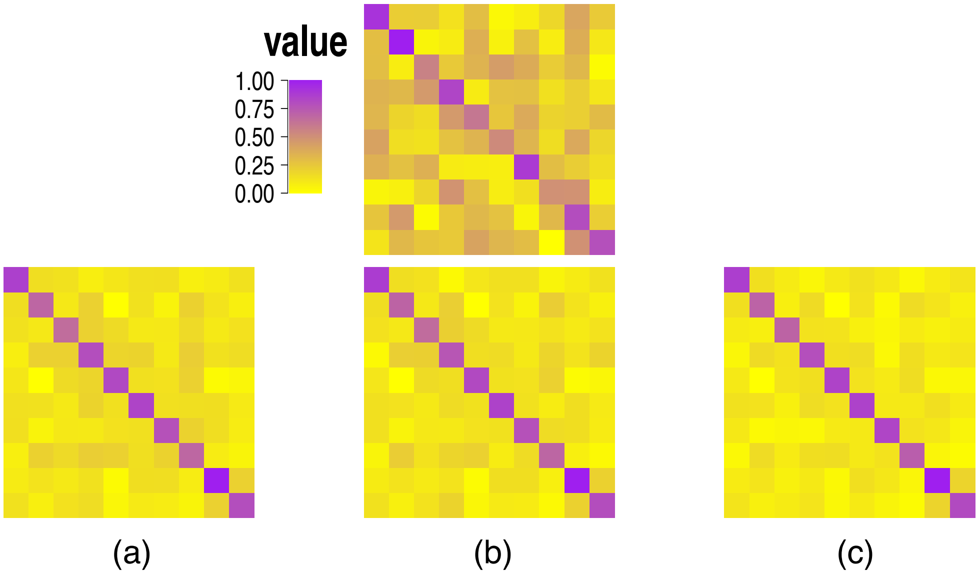

Figure S8 – Heatmaps of connectivity matrix and estimates in model 1s (heterogeneous SBM) when $K^* = K = 10$. The top heatmap shows the connectivity of the true SBM. The bottom pannels show the estimated connectivity matrices by (a) MLE, (b) VBEM, and (c) EB. All three methods have similar estimates without substantial deviation from the truth.

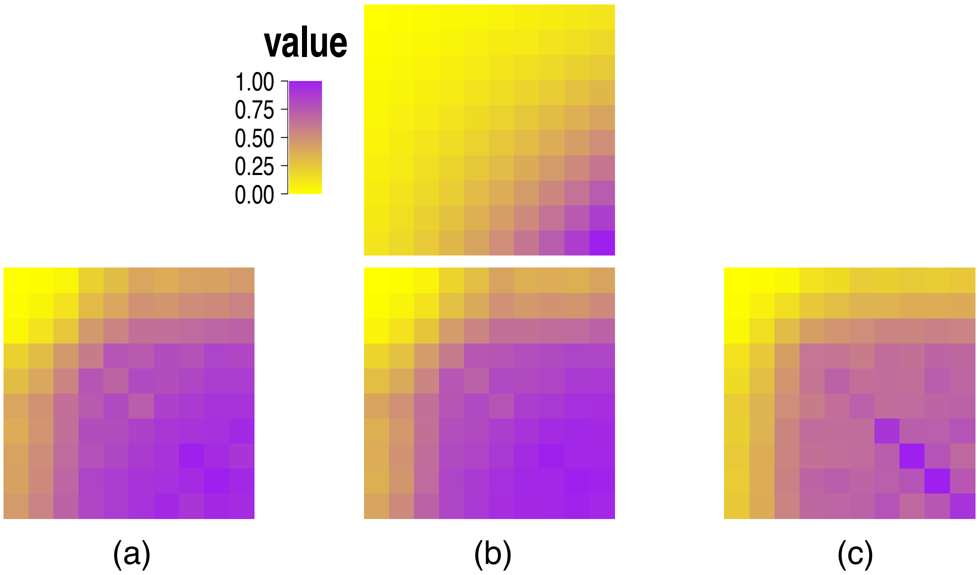

Figure S9 – Heatmaps of approximated connectivity matrix and estimates in model 3 (graphon) with $\rho = 10^{-1}$ and $\lambda = 3$. The top heatmap shows the value of the graphon function approximated by a 10×10 matrix. The bottom pannels show the estimated connectivity matrices with $K = 10$ by (a) MLE, (b) VBEM, and (c) EB. All three methods tend to overestimate the connectivity of many blocks, while EB is the one that has relatively closer values to the truth.

REFERENCES

Beal, M. and Ghahramani, Z. (2003). The variational bayesian em algorithm for incomplete data : with application to scoring graphical model structures. *Bayesian Statistics*, 7:453–464.

Daudin, J.-J., Picard, F., and Robin, S. (2008). A mixture model for random graphs. *Statistics and Computing*, 18(2):173–183.

Latouche, P., Birmele, E., and Ambroise, C. (2012). Variational bayesian inference and complexity control for stochastic block models. *Statistical Modelling*, 12(1):93–115.
