# Supplementary figures and images for: An empirical Bayes approach to stochastic blockmodels and graphons: shrinkage estimation and model selection
Source: PeerJ Comput Sci. 2022 Jul 6;8:e1006. doi: 10.7717/peerj-cs.1006 (PMC9299287; doi:10.7717/peerj-cs.1006)

Selection Criteria

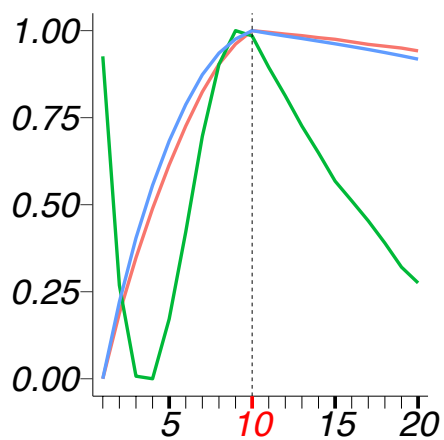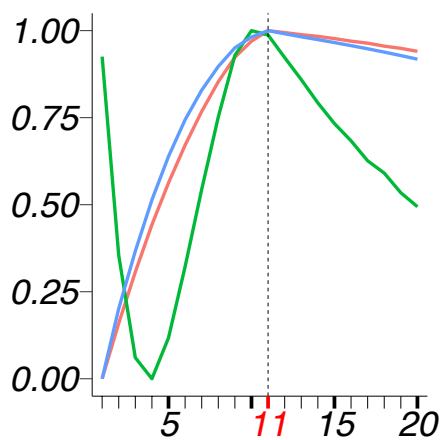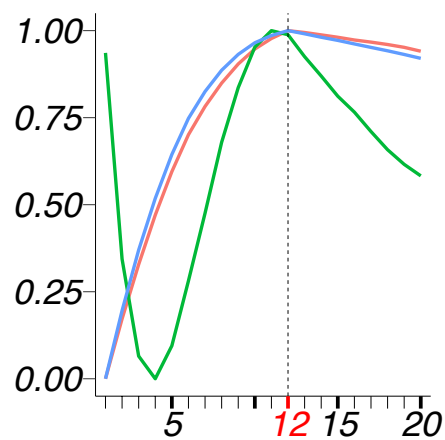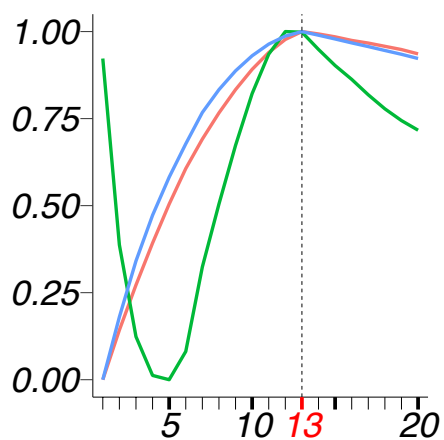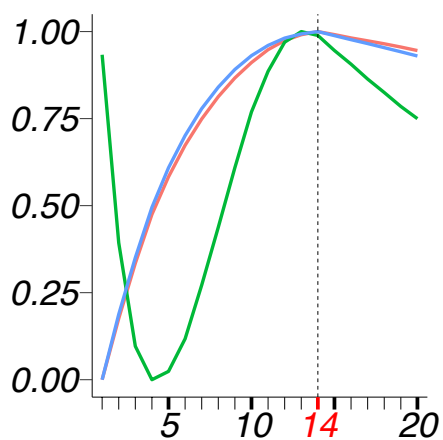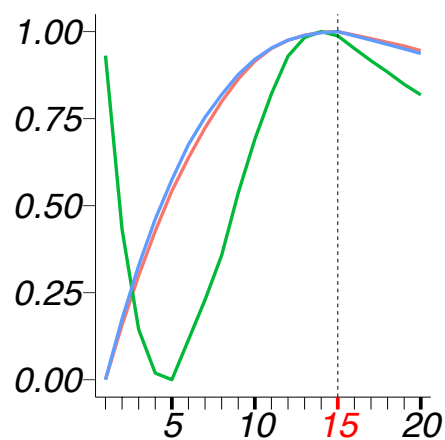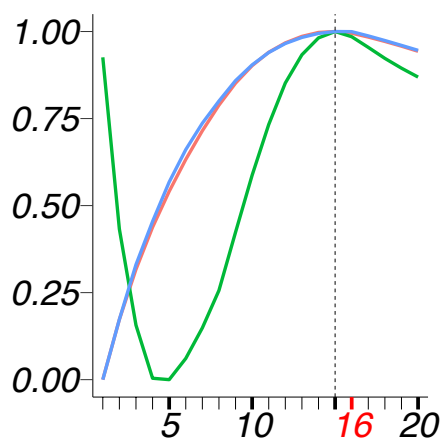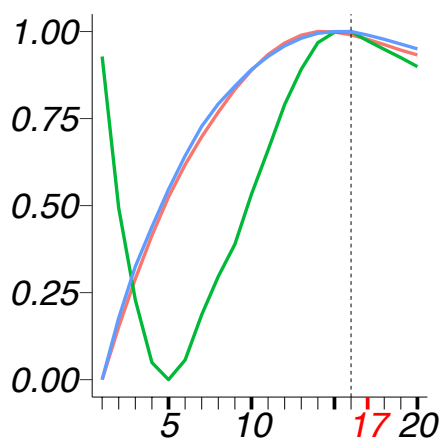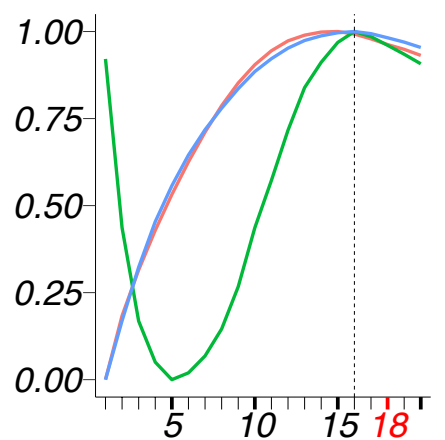

K

— CVRP

— EB

— VBEM

Supplement: Supplemental Information 2 [file peerj-cs-08-1006-s002.pdf]

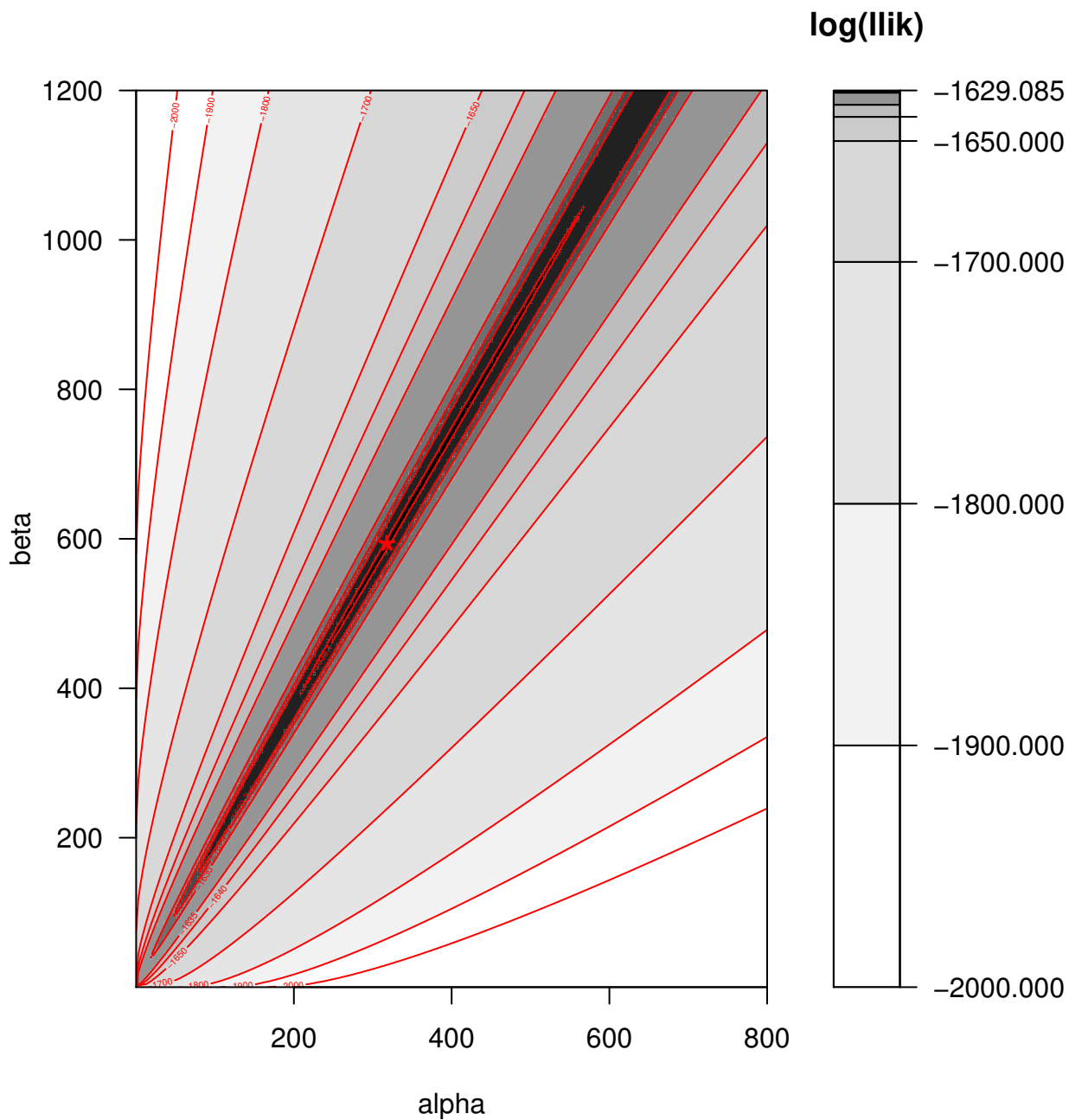

Supplement: Supplemental Information 3 [file peerj-cs-08-1006-s003.pdf]

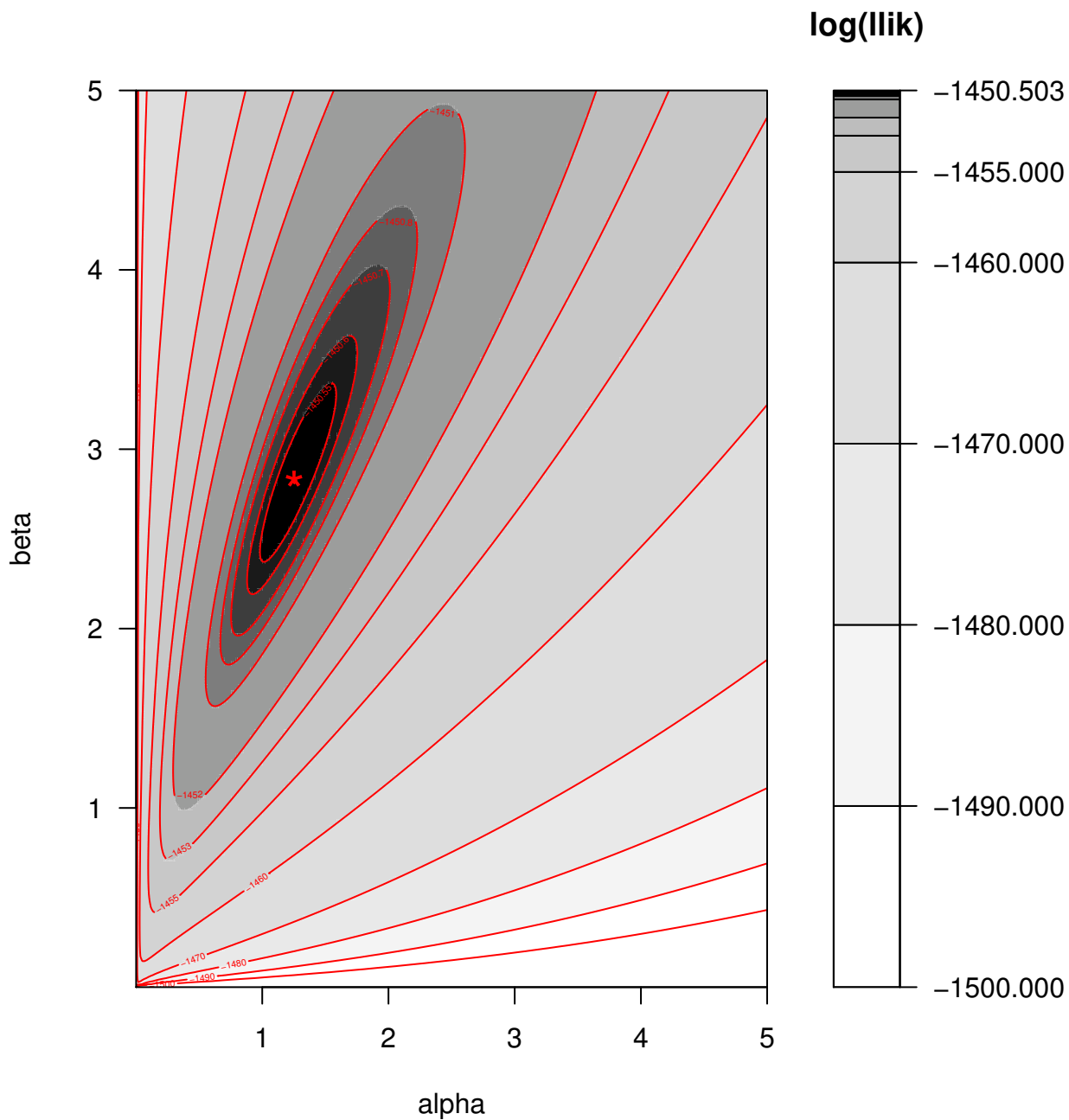

Supplement: Supplemental Information 4 [file peerj-cs-08-1006-s004.pdf]

Selection Criteria

$n = 200$

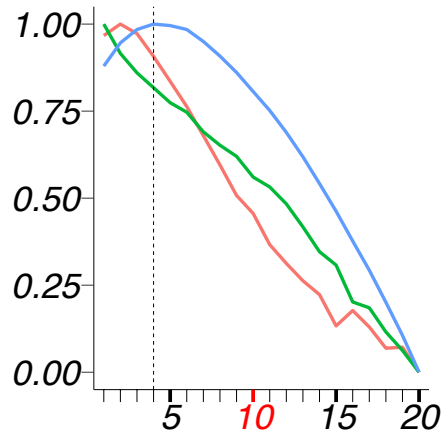

$n = 250$

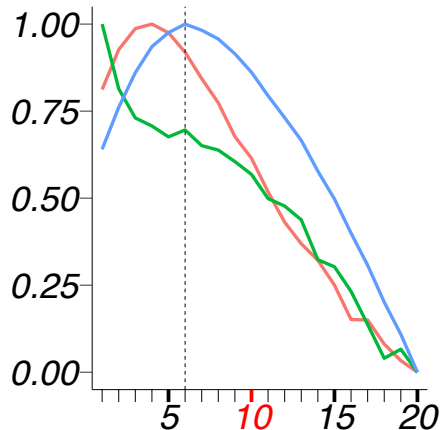

$n = 300$

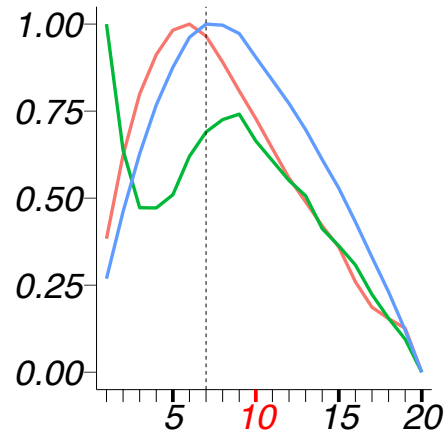

$n = 350$

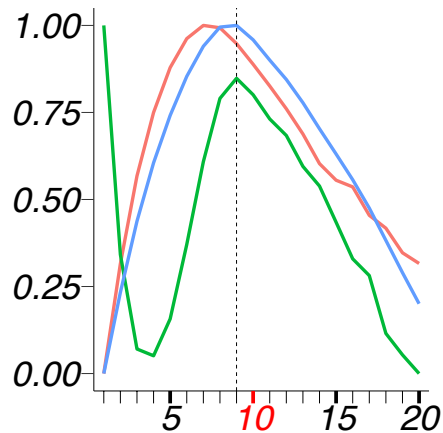

$n = 400$

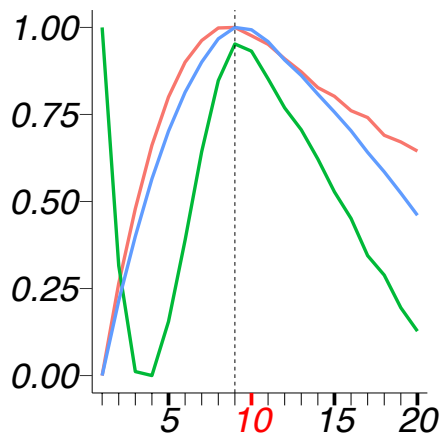

$n = 450$

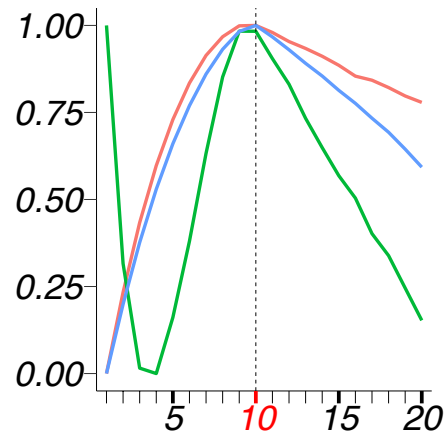

$K$

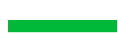

CVRP

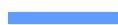

EB

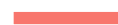

VBEM

Supplement: Supplemental Information 5 [file peerj-cs-08-1006-s005.pdf]

MSE Ratio (%)

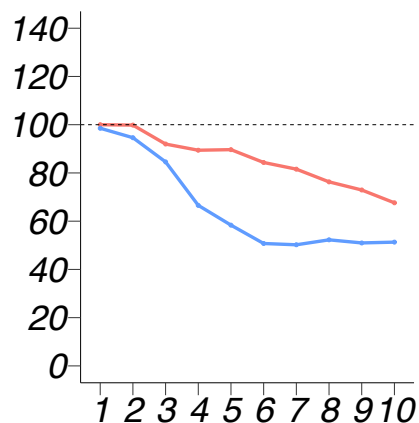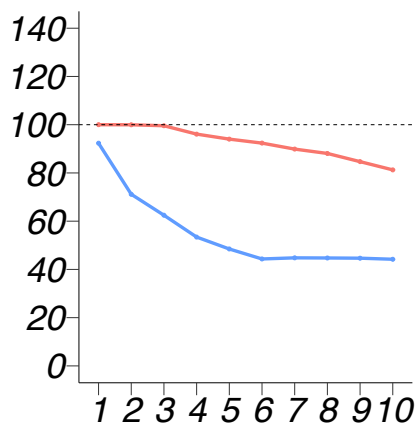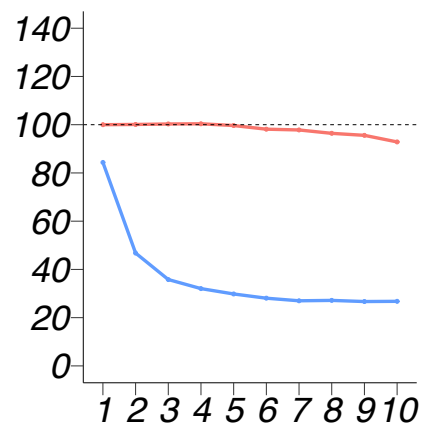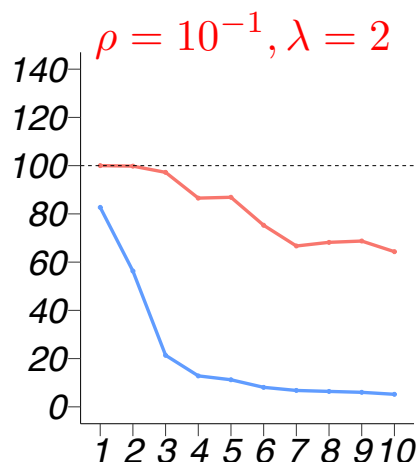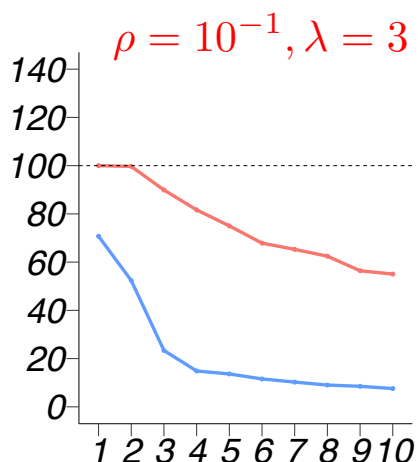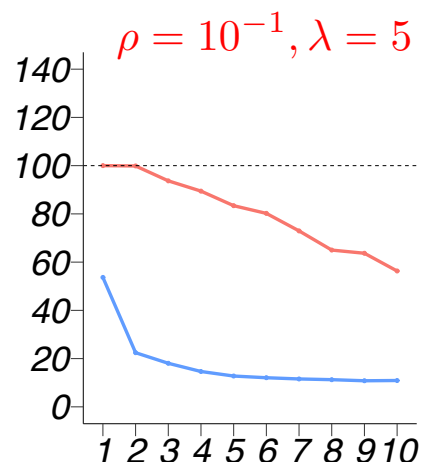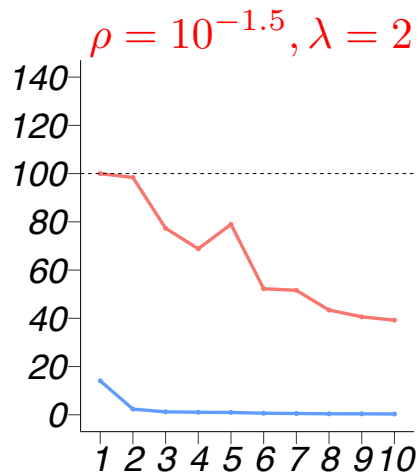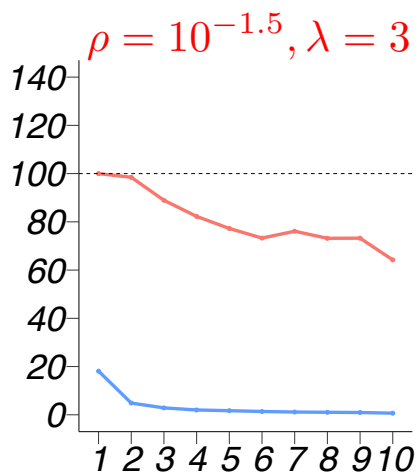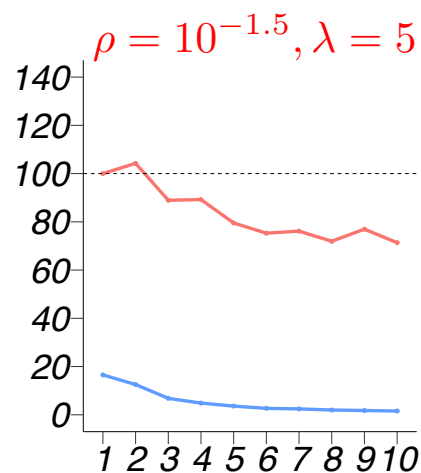

K

EB/MLE

EB/VBEM

Supplement: Supplemental Information 6 [file peerj-cs-08-1006-s006.pdf]

Selection Criteria

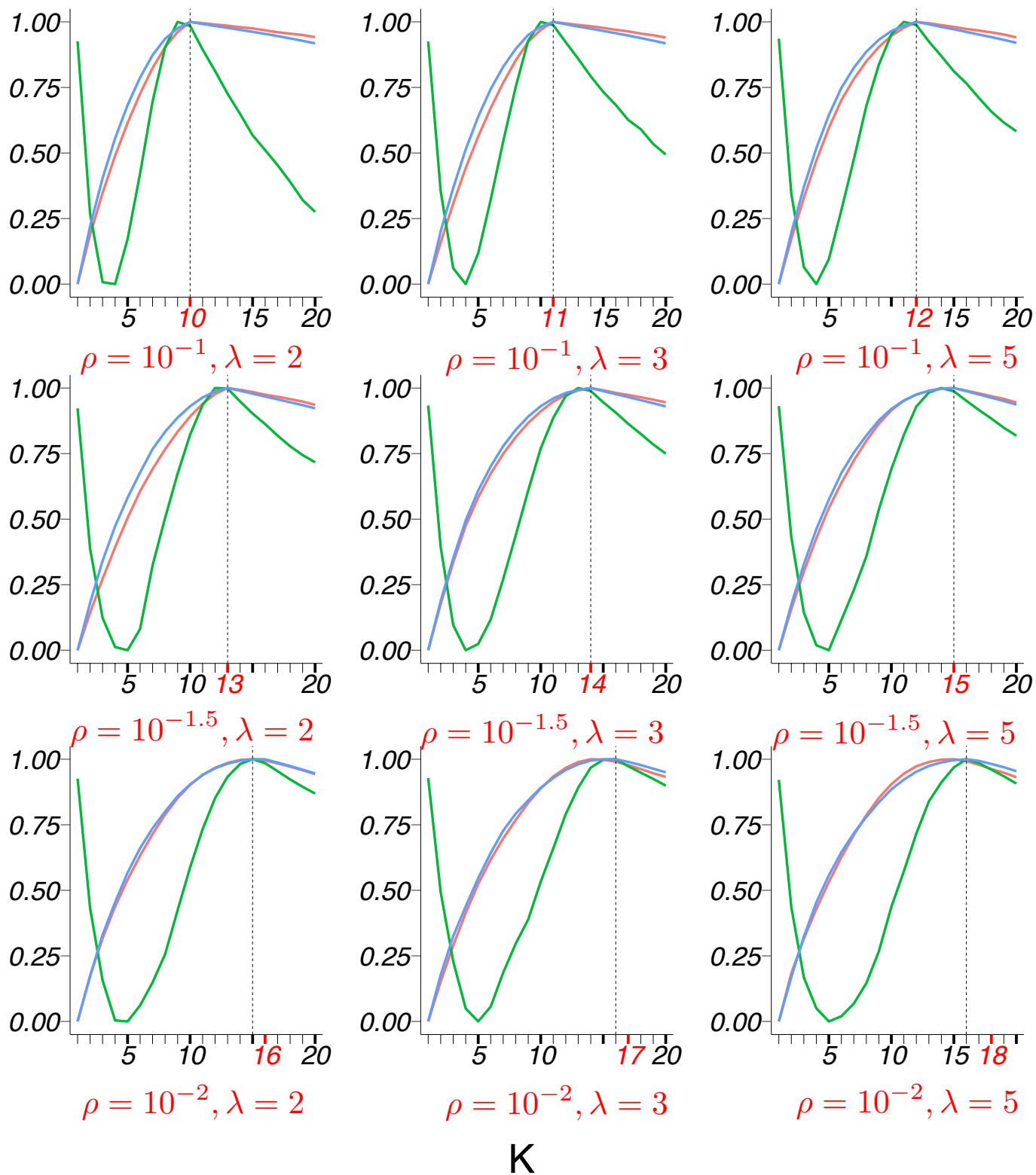

CVRP

EB

VBEM

Supplement: Supplemental Information 7 [file peerj-cs-08-1006-s007.pdf]

Selection Criteria

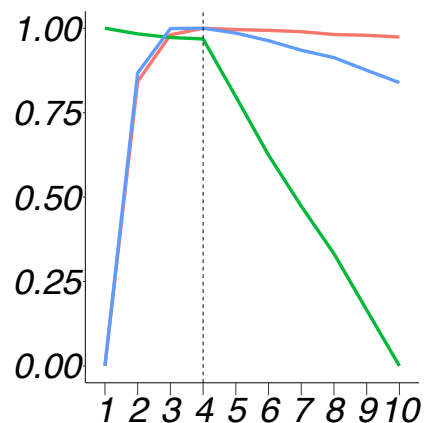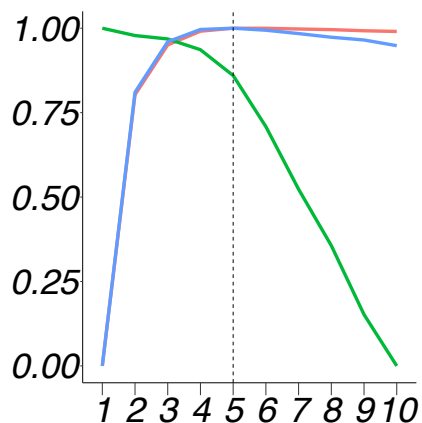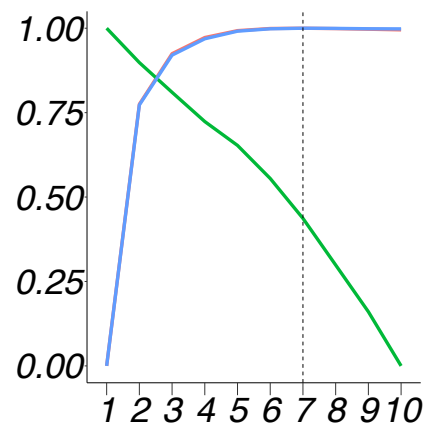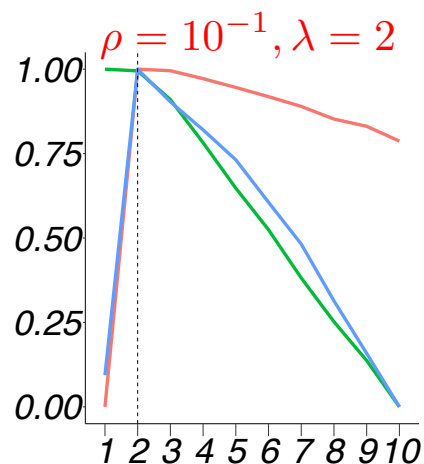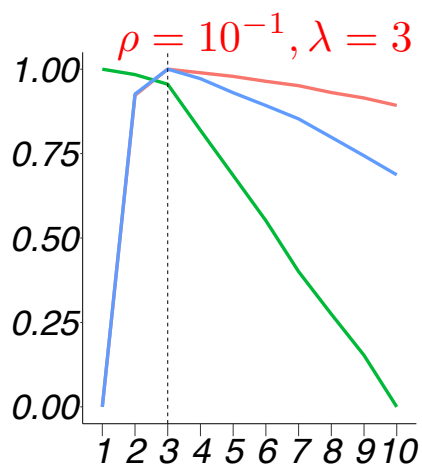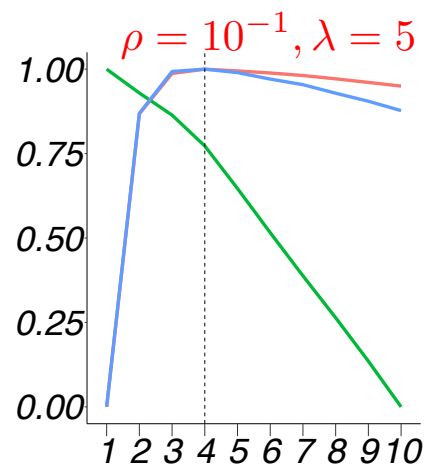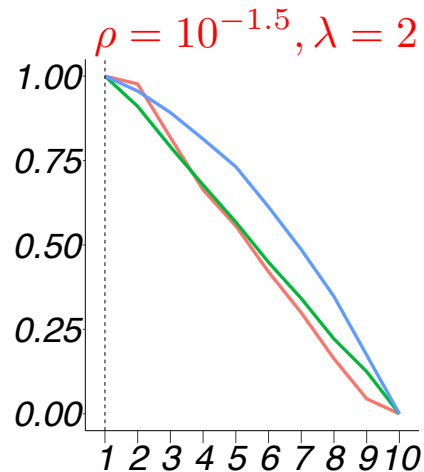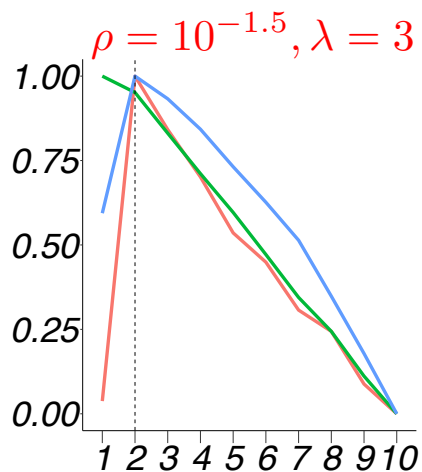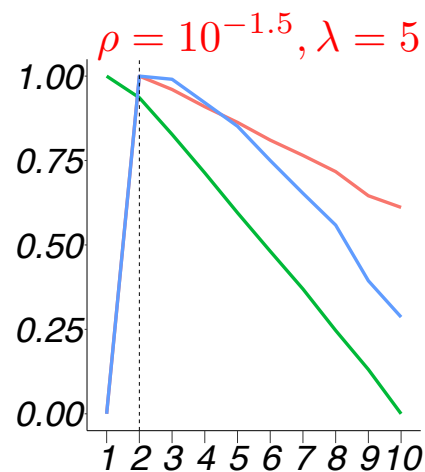

K

CVRP

EB

VBEM

Supplement: Supplemental Information 8 [file peerj-cs-08-1006-s008.pdf]

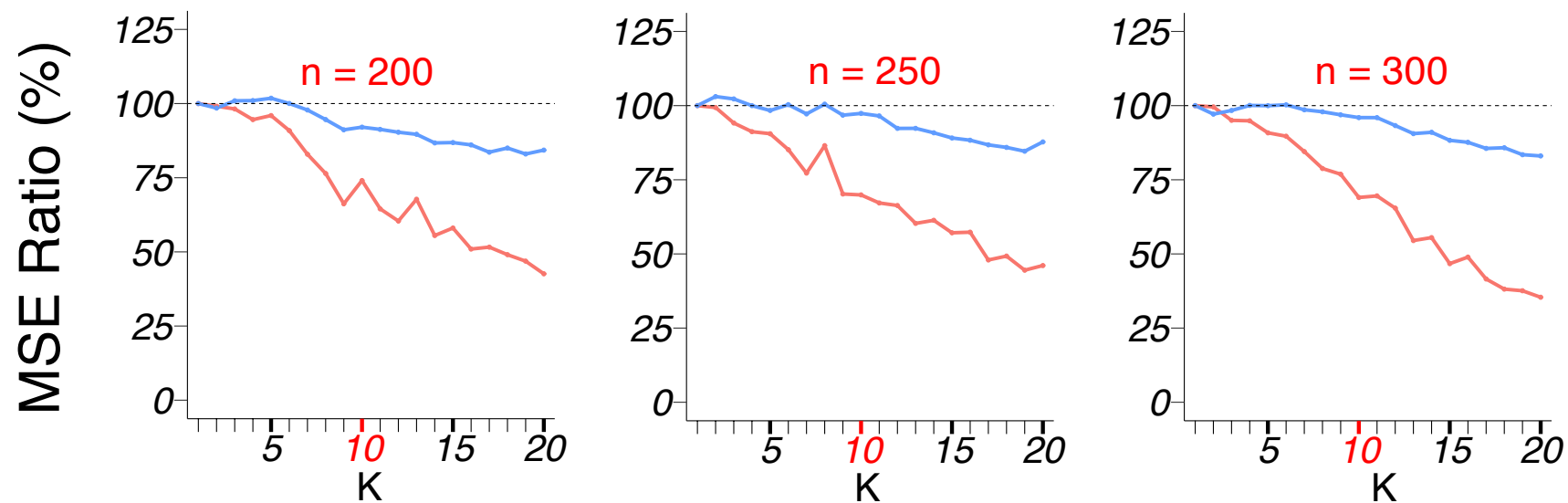

(a)

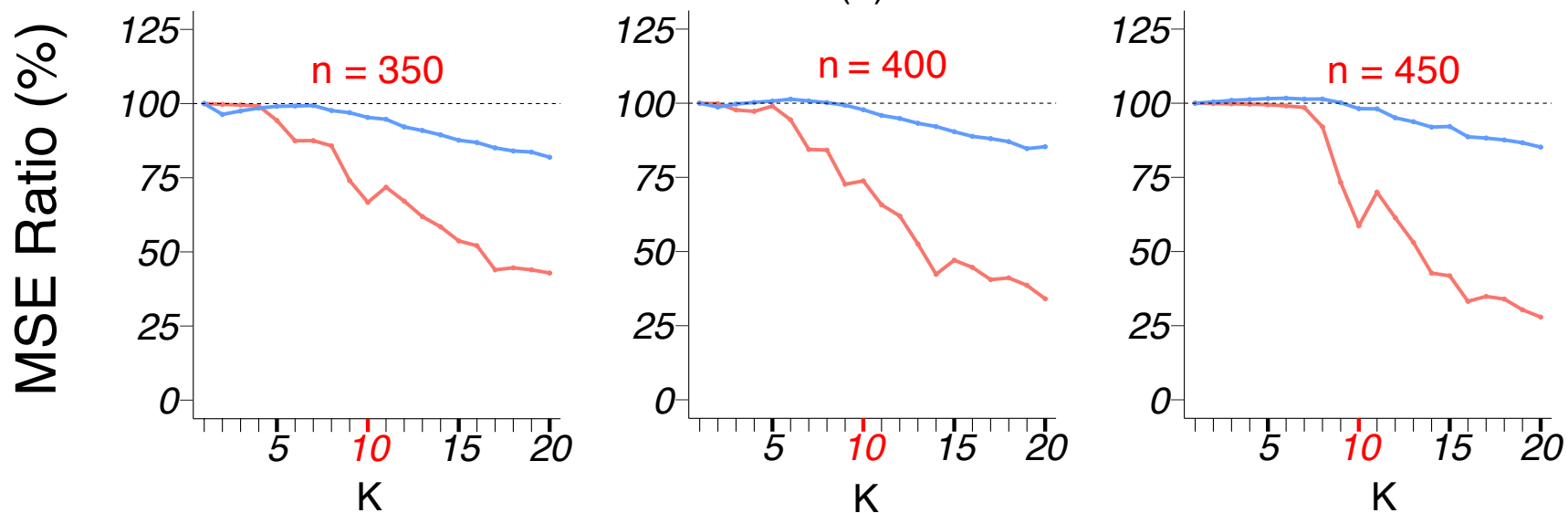

(b)

—●— EB/ML      —●— EB/VBEM

Supplement: Supplemental Information 9 [file peerj-cs-08-1006-s009.pdf]

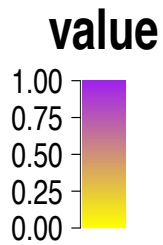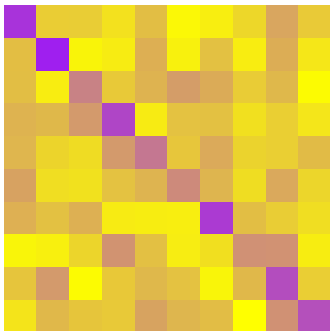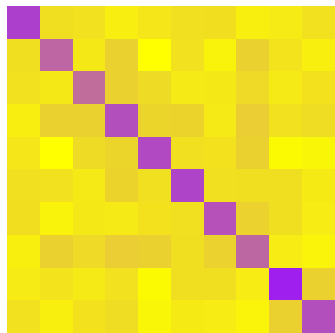

(a)

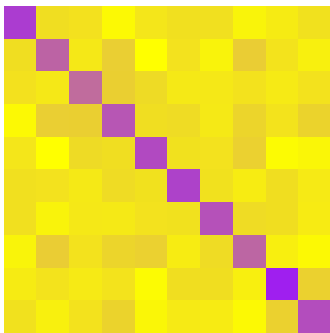

(b)

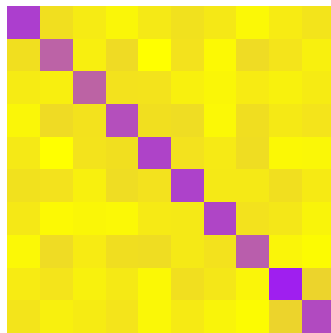

(c)

Supplement: Supplemental Information 10 [file peerj-cs-08-1006-s010.pdf]

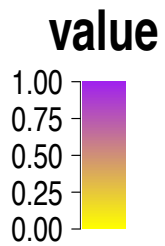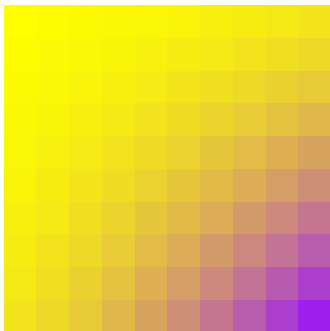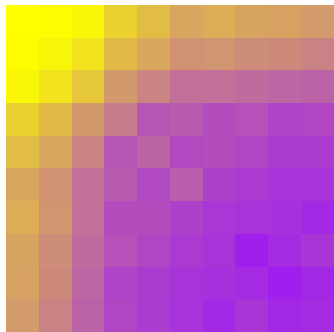

(a)

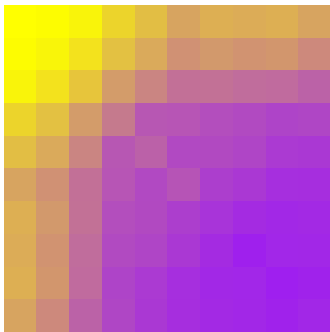

(b)

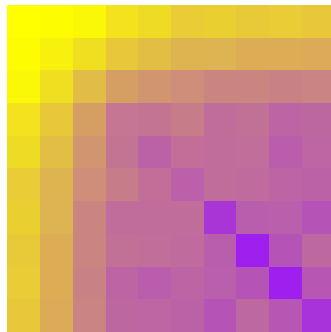

(c)

Supplement: Supplemental Information 11 [file peerj-cs-08-1006-s011.pdf]
